# Cold‐induced fibrosis and metabolic remodeling in the turtle (*Trachemys scripta*) ventricle

**DOI:** 10.1111/apha.70026

**Published:** 2025-03-15

**Authors:** Adam N. Keen, James C. McConnell, John J. Mackrill, John Marrin, Alex J. Holsgrove, Janna Crossley, Alex Henderson, Gina L. J. Galli, Dane A. Crossley, Michael J. Sherratt, Peter Gardner, Holly A. Shiels

**Affiliations:** ^1^ Division of Cardiovascular Sciences, Faculty of Biology, Medicine, and Health University of Manchester Manchester UK; ^2^ Manchester Institute of Biotechnology, University of Manchester Manchester UK; ^3^ Division of Cell Matrix Biology and Regenerative Medicine, Faculty of Biology Medicine and Health University of Manchester Manchester UK; ^4^ Department of Physiology University College Cork Cork Ireland; ^5^ Department of Biology University of North Texas Denton Texas USA; ^6^ Department of Chemical Engineering Photon Science Institute, University of Manchester Manchester UK; ^7^ Present address: Wellcome‐Wolfson Institute of Experimental Medicine Queen's University Belfast Belfast UK; ^8^ Present address: North‐West Genomic Laboratory Hub Manchester Centre for Genomic Medicine, Manchester University NHS Foundation Trust Manchester UK

**Keywords:** atomic force microscopy, collagen, FTIR, glycolysis, heart, metabolism, seasonal biology

## Abstract

**Aim:**

Cardiac fibrosis contributes to systolic and diastolic dysfunction and can disrupt electrical pathways in the heart. There are currently no therapies that prevent or reverse fibrosis in human cardiac disease. However, animals like freshwater turtles undergo seasonal remodeling of their hearts, demonstrating the plasticity of fibrotic remodeling. In *Trachemys scripta*, cold temperature affects cardiac load, suppresses metabolism, and triggers a cardiac remodeling response that includes fibrosis.

**Methods:**

We investigated this remodeling using Fourier transform infrared (FTIR) imaging spectroscopy, together with functional assessment of muscle stiffness, and molecular, histological, and enzymatic analyses in control (25°C) *T. scripta* and after 8 weeks of cold (5°C) acclimation.

**Results:**

FTIR revealed an increase in absorption bands characteristic of protein, glycogen, and collagen following cold acclimation, with a corresponding decrease in bands characteristic of lipids and phosphates. Histology confirmed these responses. Functionally, micromechanical stiffness of the ventricle increased following cold exposure assessed via atomic force microscopy (AFM) and was associated with decreased activity of regulatory matrix metalloproteinases (MMPs) and increased expression of MMP inhibitors (TMPs) which regulate collagen deposition.

**Conclusions:**

By defining the structural and metabolic underpinnings of the cold‐induced remodeling response in the turtle heart, we show commonalities between metabolic and fibrotic triggers of pathological remodeling in human cardiac disease. We propose the turtle ventricle as a novel model for studying the mechanisms underlying fibrotic and metabolic cardiac remodeling.

## 1 INTRODUCTION

Temperature is a powerful modulator of cardiac function in ectotherms and is known to induce a cardiac remodeling response in both active and passive properties. In freshwater turtles (e.g., *Trachemys scripta*), the immediate response to reduced temperature is a bradycardia with reduced twitch force, reduced ventricular power output, and reduced total cardiac output.^1^, ^2^ Stroke volume is maintained during cooling and, together with increased blood viscosity, causes an increased hemodynamic load on the heart^3^, ^4^, ^5^ that activates compensatory remodeling of the active and passive properties. The turtle heart shows a compensatory increase in muscle twitch force and maximal isometric force during prolonged cold exposure,^5^, ^6^, ^7^, ^8^ however, unlike other vertebrates, heart and ventricular mass do not increase in the turtle.^7^ Instead, cardiac output is maintained by modulating stroke volume via an increased sensitivity to cardiac preload through the Frank‐Starling response.^1^, ^9^, ^10^

Cold‐induced remodeling of passive cardiac properties has received less attention in turtles but may be equally important in ensuring adequate function. We and others have previously shown cold acclimation is associated with reduced cardiac compliance and increased fibrosis in the fish ventricle,^9^, ^11^ and with increased compliance of the vasculature and decreased compliance of the ventricle compensating for reduced systolic function in vivo at low temperatures in turtles.^9^ These findings are significant as decreases in cardiac compliance in mammals are usually pathological and associated with diastolic disfunction.^12^ Thus, we focused this study on passive remodeling of the turtle heart to probe its utility as a model for fibrotic cardiac remodeling.

Altered metabolism is a key strategy for surviving prolonged cold and preparing for winter hypoxia in turtles.^13^, ^14^, ^15^ During winter or following cold acclimation, many species of freshwater turtles enter a state of periodic inactivity characterized by reduced metabolic requirements, allowing turtles to rely on glycolysis for ATP production, fueled by glycogen stores in the liver.^16^, ^17^, ^18^, ^19^, ^20^, ^21^, ^22^ Glycolysis slows the ATP production rate but improves myocardial oxygen efficiency per unit of ATP produced.^23^ The Q_10_‐dependent effect of temperature on metabolic rate means that the reduced rate of ATP production from glycolysis is sufficient to sustain myocardial function over prolonged periods. Metabolic changes are acknowledged as key triggers in the pathogenic process of cardiac fibrosis in humans, and metabolically targeted therapies are of growing interest as a potential strategy for fibrosis prevention and reduction.^24^

Here, we take an integrative approach to understand the biochemical, physiological, structural, and metabolic remodeling of *T. scripta* myocardium in response to prolonged cold temperatures, with a focus on the passive properties of the heart to test its utility as a new fibrosis model. We used Fourier transform infrared (FTIR) imaging spectroscopy to holistically analyze the biochemistry of cardiac tissue from control (25°C) and cold (5°C)‐acclimated turtles. FTIR provides a novel way to assay biological tissue for subtle differences in molecular structure due to unique stretching and bending vibrations of functional groups at discrete infrared wavelengths.^25^, ^26^, ^27^, ^28^, ^29^, ^30^ We have recently shown that FTIR can be used to detect subtle variances in the cardiac tissue of thermally acclimated fish.^31^ To assess changes in the passive mechanical properties of the heart following cold acclimation, we use atomic force microscopy (AFM) indentation and support this work with assessment of extracellular matrix (ECM) proteins and gene expression via histology, gelatin zymography, and qPCR. To assess changes in metabolic substrate utilization, we used FTIR in combination with histology to assess lipids, and histology and biochemical assays to assess glycogen content. Our results provide the most comprehensive analyses we know of to date, detailing the remodeling of the passive properties of the turtle heart with cold. The commonalities between metabolic alterations and fibrotic deposition suggest the turtle heart may provide a unique model for understanding the mechanisms associated with fibrotic remodeling in human hearts.

## 2 MATERIALS AND METHODS

### 2.1 Experimental animals and acclimation

Adult, male and female red‐eared slider turtles (*Trachemys scripta*; *n* = 5 for each group; mean body mass = 1352 ± 69 g) were wild‐caught from the Lake Lewisville drainage in the Dallas Fort Worth area from Army Corp of Engineer's land as allowed by Texas Parks and Wildlife Scientific Research Permit (SPR‐1114‐257) and transported to the University of North Texas. Here, they were housed in 50 L plastic containers (dimensions 50:50:100 cm) containing freshwater at a temperature of 25 ± 0.3°C on a 12:12‐h light–dark cycle. Water quality was maintained by 100% water changes twice a week, and all animals were fed three times per week on commercial reptile feed (Aquatic turtle diet, Mazuri exotic animal nutrition). After 2 weeks, 5 turtles were randomly assigned for cold acclimation, where ambient temperature was reduced by 1°C per day until 5 ± 0.3°C was reached, and turtles were maintained at this temperature for a minimum of 8 and a maximum of 10 weeks before experimentation. The acclimation temperatures were chosen based on summer and winter conditions in the area. A minimum acclimation period of 8 weeks was chosen to align with our previous studies on fish where 8 weeks resulted in cardiovascular structural remodeling.^2^, ^6^, ^9^ Animals selected for cold acclimation were fasted following temperature reduction. Temperatures were maintained via walk‐in temperature‐controlled rooms (model IR‐912 L5; Percival Scientific, Perry, IA) and animals were maintained in water without an area for basking. All animals survived the acclimation protocols, and there were no signs of poor health in either group. Animal care adhered to the University of North Texas animal care and use protocol (IACUC #11‐007).

### 2.2 Tissue processing

Animals were killed by the administration of a lethal dose of sodium pentobarbital (150 mg kg^−1^) into the forelimb, and the heart was excised within 5 min from the time of injection. Prior to euthanasia, the hearts were treated with 2,3‐Butanedione monoxime (BDM), a myosin inhibitor, to ensure the tissue was in a relaxed state before removal. The heart was rinsed in phosphate‐buffered saline (PBS) which is calcium‐free, and mass was taken. The ventricle was dissected from the atria, weighed, and bisected down the sagittal plane, with one half snap frozen in OCT (Thermo Fisher Scientific, Waltham, MA, USA) within a mold made with aluminum foil, by immersion in liquid nitrogen cooled 2‐methylbutane (Sigma‐Aldrich, St. Louis, MO, USA) and stored at −80°C. The other half was fixed in 10% neutral buffered formalin solution (Sigma‐Aldrich, St. Louis, MO, USA), processed, and embedded in paraffin wax so that sections could be cut in the transverse/axial plane. All sections were taken from the middle 50% of the ventricle, and serial sections were used across experiments where possible. Efforts were made to standardize the region of the ventricle from which measurements were made between hearts and analyses.

### 2.3 Fourier transform infrared (FTIR) imaging spectroscopy

Frozen ventricle was sectioned at 5 μm (Leica CM3050S cryostat, Leica, Wetzlar, Germany), mounted on calcium fluoride (CaF_2_) slides, and stored at −80°C. Frozen tissue was thawed in a vacuum desiccator for 60 min prior to being placed in the purge box of the spectrometer. Transmission mode FTIR imaging spectroscopy was performed on a Cary670‐IR spectrometer coupled with a Cary 620‐IR imaging microscope (Agilent Technologies, CA, USA) equipped with a 128 × 128 pixel liquid nitrogen cooled Mercury‐Cadmium‐Telluride focal plane array (FPA) detector. Data were collected in the 950–3800 cm^−1^ range at a spectral resolution of 5 cm^−1^, with the co‐addition of 96 scans for sample spectra and 256 scans for background spectra. At a pixel size of 5.5 μm, a tissue sampling area of 1500 μm × 1500 μm was captured by the hyperspectral image. Sample areas were within an area of the tissue corresponding to 25–75% of the radius of the tissue section, so that they were not too close to either the pericardium or lumen of the ventricle, thus falling within the trabeculated region of the heart.

### 2.4 Atomic force microscopy (AFM)

Frozen ventricle was sectioned at 5 μm (Leica CM3050S cryostat, Leica, Wetzlar, Germany) and mounted onto glass slides (Super frost plus, ThermoFisher Scientific, Waltham, MA, USA). Excess OCT was removed with distilled water, and the slides were left to dry for ~12 h. This methodology is consistent with previous work^32^, ^33^ which found tissue sections are best preserved dehydrated, with rehydration performed when nanomechanical measurements are required. Nano‐indentation was carried out using a Bioscope Catalyst AFM (Bruker, Coventry, UK) mounted onto an Eclipse T1 inverted optical microscope (Nikon, Kingston, UK) fitted with a spherically tipped cantilever (nominal radius and spring constant of 1 μm and 3 Nm^−1^ respectively; Windsor Scientific Ltd., Slough, UK) running Nanoscope Software v8.15 (Bruker, Coventry, UK). The local reduced modulus (M_r_) was determined for each of 400 points in a 50 × 50 μm region, indented at a frequency of 1 Hz with lateral spacing of 2.5 μm. The extend curve was used in conjunction with a contact point‐based model to calculate the M_r_ for each indentation.^34^ For each biological sample, 400 force curves were collected at three distinct 50 μm^2^ regions. The regions surveyed were within an area of the tissue corresponding to 25–75% of the radius of the tissue section and, therefore, the trabeculated region of the heart. Once all 400 force curves had been generated, quality control was applied whereby any force values falling more than two standard deviations away from the mean value were discarded to account for failed indents.^35^ Data loss at this stage was <10% (data not shown).

### 2.5 Histological staining

For collagen analyses, formalin‐fixed and paraffin‐embedded ventricular samples were sectioned at 5 μm and mounted onto glass slides. Sections were stained with picro‐sirus red to show fibrillar collagen^36^ and imaged under bright‐field and plane‐polarized light (Leica, Wetzlar, Germany). Quantitative analyses of collagen orientation (coherency) were conducted on picro‐sirus red stained polarized micrographs using the OrientationJ plugin on ImageJ.^35^, ^37^, ^38^ Three tissue sections were considered for each individual.

To assess lipid content of the tissue, frozen ventricular tissue was sectioned at 5 μm, mounted onto glass slides, and stained with oil red‐O stain for lipid. A negative control was prepared for each section by taking serial sections and treating them with acetone prior to the oil red‐O protocol to remove all lipid. To assess glycogen content of the tissue, formalin‐fixed tissue samples were processed, embedded in paraffin wax, sectioned at 5 μm mounted onto glass slides, and stained with Periodic acid‐Schiff (PAS) stain for glycogen. Negative controls were prepared for each section by taking serial sections and digesting the glycogen with amylase before the PAS staining protocol. Images were analyzed using bright‐field microscopy (Leica Wetzlar, Germany) and ImageJ software.^35^ Lipid and glycogen content of the tissues were determined by pixel count compared to total tissue and then expressed as a percentage increase compared to that section's control. For all histological analyses, three tissue sections were considered for each individual, taken from the central 50% of the embedded tissue. On each tissue section, 3 separate image montages were taken along transects across the full diameter of the cross section. All histological analyses were conducted blind to the acclimation group.

### 2.6 MMP gelatin zymography

To characterize the abundance and activation of MMPs, we used SDS‐PAGE‐based gelatin zymography. Snap‐frozen ventricular tissue was rinsed with phosphate‐buffered saline; then, protein was extracted in ten volumes per wet weight of 0.05% Brij‐35, 10 mM CaCl_2_, and 50 mM Tris–HCl pH 7.4 on ice using three 10s bursts of an MSE Soniprep150 sonicator (exponential probe, 10 μm amplitude). Extracts were cleared by centrifugation at 10 000 *g* for 10 min, and protein content was determined using the Bradford assay with bovine serum albumin as a standard. Equal quantities of protein (1 μg/lane) were analyzed by gelatin SDS‐PAGE, as described by Lødemel et al.^39^ Conditioned media from HepG2 cell cultures (100 ng protein/lane) and recombinant active human MMP‐2 (1 ng protein/lane, Millipore) were used as positive controls. The abundance of each gelatinase band was measured using the ‘Gel’ function of ImageJ.

### 2.7 In situ MMP gelatin zymography

The activity of endogenous MMP gelatinase was semi‐quantitatively analyzed by in situ zymography of tissue cryosections, following previously published methodology.^40^, ^41^ Frozen tissue was sectioned at 10 μm and mounted onto glass slides, and incubated with low‐temperature gelling agarose (Sigma‐Aldrich, St. Louis, MO, USA), DQ gelatin (0.1 mg mL^−1^; porcine; Invitrogen, Thermo Fisher Scientific, Waltham, MA, USA) and DAPI (1 μg mL^−1^) for 1 h at 4°C and then 18 h at room temperature. Slides were fluorescently imaged using a FITC filter (Leica, Wetzlar, Germany). To remove any effect of tissue auto‐fluorescence, negative control slides were used to determine the microscope settings for each section. Three tissue sections were considered for each individual. On each tissue section, 3 separate image montages were taken along transects across the full diameter of the cross section. Following background subtraction, mean fluorescence intensity was calculated for each image using ImageJ. Analysis was conducted blind to the acclimation group, and tissue sections were taken from the central 50% of the embedded ventricle.

### 2.8 Quantitative reverse transcription PCR (RT‐qPCR)

Transcript abundance of genes associated with collagen I (COL1α2), matrix metalloproteinases (MMP2 and MMP9), a tissue inhibitor of metalloproteinases (TIMP2) and GAPDH was quantified in the ventricles of cold‐acclimated and control turtles (*n* = 5 ventricles for each temperature) using RT‐qPCR, following the standard methodology for RNA extraction. Briefly, forward and reverse primers for the genes of interest were designed using Primer 3 from mRNA sequences available on PubMed (specific marker genes and primers are in Table 1) and purchased from Integrated DNA Technologies (Coralville, IA, USA). Total RNA was extracted from each ventricle sample in RNAzol RT (Molecular research center Inc.) following the manufacturer's suggested protocol for total RNA extraction. Briefly, frozen tissue was placed in a pre‐cooled mortar, and RNAzol RT was added to the sample at a ratio of 1 mL per 100 mg of tissue. The tissue was then ground and processed as previously described.^41^ RNA was then precipitated with isopropyl alcohol. The concentration and purity of total RNA were measured using a NanoDrop™‐1000 spectrophotometer (Thermo Scientific, Wilmington, DE, USA). All samples had 260/280 absorbance ratios of 1.7–2.0 and displayed discrete 18S and 28S rRNA bands on agarose gels. cDNA was synthesized by reverse transcribing 1 μg of total RNA in a 20 μL reaction using iScript reverse transcription super mix as per the manufacturer's protocol (BioRad, Hercules CA). Real‐time PCR was completed using 5 μL of 2× SsoFast™ EvaGreen® Supermix, 0.3 μL of forward primer, and 0.3 μL of reverse primer (final primer concentration = 300 nM), 2 μL of heart cDNA (equivalent to 10 ng of input RNA), and water to bring the total volume to 10 μL. Reactions were run on white 384‐well plates in a CFX384 Real‐Time PCR Detection System (BioRad). The thermal profile was 95°C for 30 s to activate DNA polymerase, followed by 40 cycles of two‐step PCR (95°C for 5 s and 60°C for 5 s). Negative control reactions (no reverse transcriptase controls and water controls) demonstrated no contamination with genomic DNA or exogenous PCR products. GAPDH was used as a temperature‐insensitive control and showed no difference in transcript abundance with temperature acclimation. A melt curve at the end of each run verified that a single product was amplified. Standards were made as previously described.^42^, ^43^ Standard curves were log‐linear over eight orders of magnitude, which allowed quantification of gene expression in attograms of cDNA per 1 μg of input RNA.

**TABLE 1:** The specific marker genes with primers used for quantitative reverse transcription PCR analysis.

Gene	Primer pair	GenBank accession number	Function
COL1α2	5′‐AACTTGCCTTCATGCGTCTG‐3′ 5′‐GGTTGCCAGTTTCCTCATCC‐3′	NW_007359883.1	Fibrosis
MMP2	5′‐GGTGCCCAAAAGACAACTGC‐3′ 5′‐TGTTTCAGGCAGCCCAAAGA‐3′	NC_024229.1	Inhibit fibrosis
MMP9	5′‐CGGAGGATGCAGAAGAAGCT‐3′ 5′‐TGATCCCACTTGAGGTCTCC‐3′	NW_007281435.1	Inhibit fibrosis
TIMP2	5′‐ATAGAGTTAATTTACACAGCTCCCT‐3′ 5′‐ATATTCCTTCTTCCCGCCGG‐3′	NW_007281340.1	Inhibit MMPs

### 2.9 Glycogen content

Glycogen content was determined by first converting glycogen stores into glucose and then by using the rate of 6‐phosphogluconic acid conversion (through measuring NADPH production) as an assay for glucose content. In brief, ~20 mg of ventricle from each turtle (*n* = 5) were placed in Eppendorf tubes containing 2 M HCl. Samples were boiled for 60 mins to achieve full hydrolysis of the tissue and glycogen. Samples were minced with scissors within the first 5 min and vortexed every 20 min, and then allowed to cool to room temperature. The original weights were reconstituted with ddH2O, and the hydrolysis products were neutralized with 2 M NaOH. Samples were vortexed and centrifuged for 10 mins at 22000*g*. Glucose concentration was determined using a glucose HK assay kit (sigma G2020). Hydrolyzed samples were incubated with reconstituted assay medium for 5 mins at room temperature. 0.5 mM of fresh β‐D‐glucose was used as a standard. NADPH content was measured at 384 nm. Glycogen content was determined as: AE(sample/standard) * [standard] * Vstandard * Vtotal/sample/μg of protein.

### 2.10 Calculations and statistical analyses

Infrared spectral data was imported into MATLAB (MATLAB 2014a, Mathworks, USA) and quality tested by the amide I region (1597–1738 cm^−1^). The absorbance values to determine which spectra were accepted or rejected were assessed separately for each hyperspectral image by reference to an image of the tissue section. The band associated with ambient gas‐phase CO_2_ was removed, as was any data outside of the specified wavenumber range. Data was subjected to a PCA noise reduction,^44^, ^45^ vector normalization, and RMieS‐EMSC correction with 100 iterations using a Matrigel™ spectrum as the initial reference point.^46^ Following the scatter correction, regions of interest were taken to ensure there were no artifacts due to sample preparation and that we captured the full biochemistry. The region of interest data was then subjected to a K‐fold algorithm which randomized the spectra and then condensed them into 1000 average spectra for each individual turtle. All subsequent analyses were conducted on these K‐folded spectra. Following analysis of mean spectra, we transformed the data into the second derivative to deconvolute some of the broad peaks and enhance peak resolution.

Post hoc analyses of AFM force curves were performed using Nanoscope Analysis v1.40 (Bruker, Coventry, UK), whereby a baseline correction was applied to each curve before a force fit was applied using a Herzian (spherical) model and a maximum force fit of 70%.^35^ Gene expression patterns were assessed by 2‐way ANCOVA and a Holm‐Sidak post hoc test to account for multiple comparisons. Lipid and glycogen assessment of the tissue sections via histology were determined by pixel count compared to the corresponding negative control section, using ImageJ. Differences in lipid and glycogen content, collagen coherency, MMP activity, and MMP abundance were assessed by general linear model (GLM; students *t*‐test for parametric, Mann–Whitney U for non‐parametric), with each parameter as the test variables and acclimation group as the grouping variable, using SPSS Statistics 20 (IBM, Armonk, NY, USA). For all analyses, significance was considered to be *p* < 0.05, except for atomic force curves where significance was considered at *p* < 0.005. Values are presented as mean ± *SE* throughout except for atomic force curves where values are mean ± *SD* Statistical details are provided in the figure legends.

## 3 RESULTS

### 3.1 Thermal acclimation regulates tissue biochemistry

To assess the biochemistry of turtle ventricular myocardium, we used FTIR imaging spectroscopy. As the turtle ventricle is comprised of a small compact layer and extensive spongy trabeculated regions, we first analyzed the biochemistry of control tissue sections to see if there was any biochemical heterogeneity between the layers (Figure S1). We used a combination of principal component analysis and k‐means cluster analysis to evaluate the biochemistry of the whole tissue width and specific regions of interest and did not find any significant heterogeneity (Figure S1).

**FIGURE 1:**
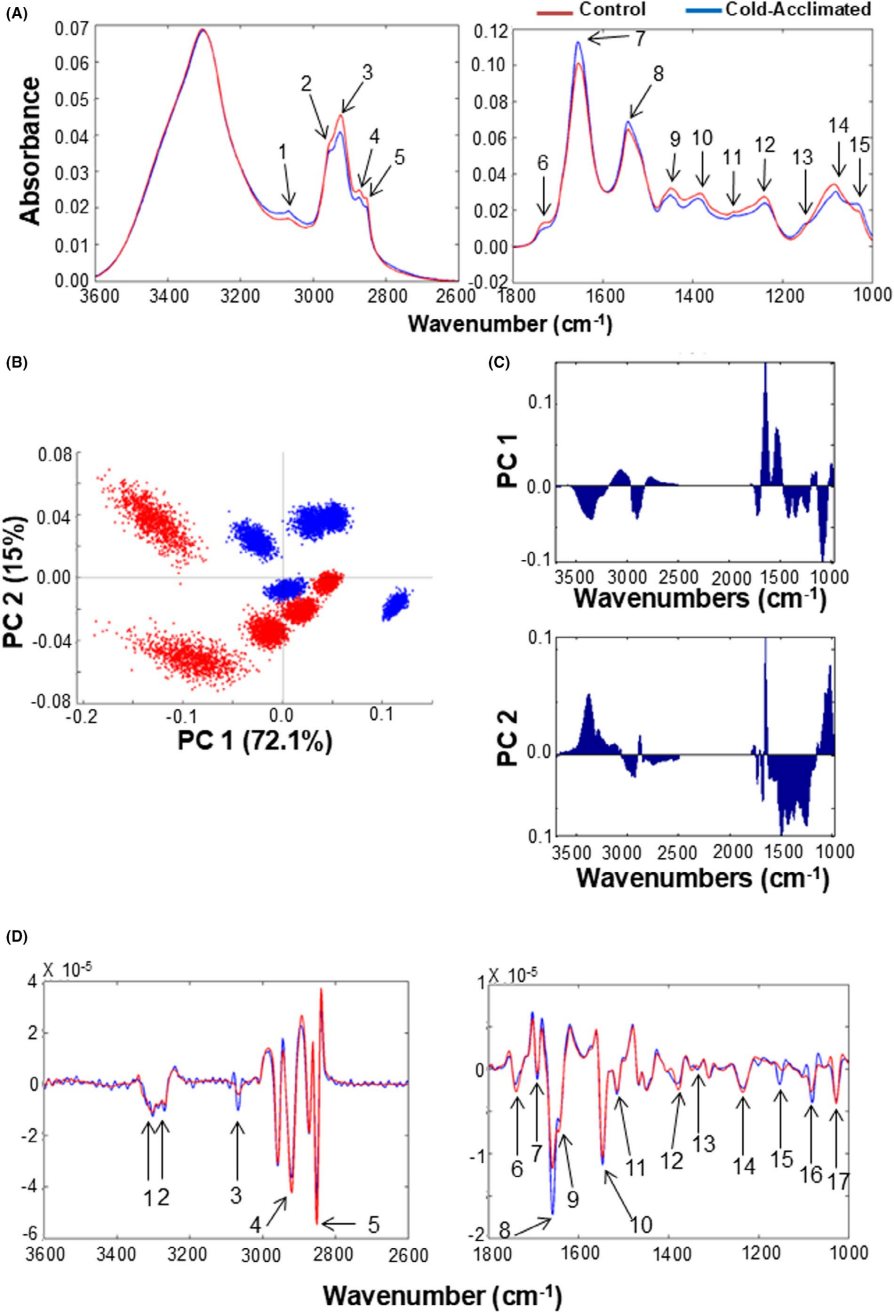
Thermal acclimation regulates tissue biochemistry. (A) Mean spectra for cold‐acclimated (5°C; blue) and control (25°C; red) turtle ventricle cryosections (*n* = 5, for each group). Spectra ‐were quality tested and subjected to noise reduction, vector normalization and RMieS scatter correction. Numbers denote key spectral bands of interest. 1, 3069 cm^−1^; 2, 2953 cm^−1^; 3, 2924 cm^−1^; 4, 2872 cm^−1^; 5, 2853 cm^−1^; 6, 1736 cm^−1^; 7, 1655; cm^−1^ 8, 1545 cm^−1^; 9, 1451 cm^−1^; 10, 1387 cm^−1^; 11, 1308 cm^−1^; 12, 1240 cm^−1^; 13, 1152 cm^−1^; 14, 1086 cm^−1^; 15, 1034 cm^−1^, for which the functional groups are listed in Table 2. (B) Principal component (PC) scores plot for PC 1 and PC 2 for cold‐acclimated and control tissue. (C) The corresponding PC loadings plot for PC 1 and PC 2. In the loadings plot, positive values show the key characteristics that are increased in that particular PC and negative values show the key characteristics that are reduced in that particular PC. Spectra from each individual turtle have been subjected to a K‐fold algorithm which reduces the spectral number to 1000 mean spectra per individual. (D) The second derivative of mean spectra for cold‐acclimated (blue) and control (red) turtle ventricle cryosections. Numbers denote key spectral bands of interest. 1, 3302 cm^−1^; 2, 3270 cm^−1^; 3, 3067 cm^−1^; 4, 2920 cm^−1^; 5, 2851 cm^−1^; 6, 1738 cm^−1^; 7, 1692 cm^−1^; 8, 1657 cm^−1^; 9, 1645 cm^−1^; 10, 1547 cm^−1^; 11, 1514 cm^−1^; 12, 1377 cm^−1^; 13, 1338 cm^−1^; 14, 1235 cm^−1^; 15, 1152 cm^−1^; 16, 1080 cm^−1^; 17, 1029 cm^−1^.

As the tissue sections showed biochemical homogeneity across the ventricle, we used hyperspectral data from the whole image area to compare between control and cold‐acclimated groups. The differences in infrared absorption between acclimation groups across key spectral bands are detailed in Table 2. Following cold acclimation, there was an increase in infrared absorbance peaks correlating with amide B, amide I, and amide II, mean centered at 3069 cm^−1^, 1655 cm,^−1^ and 1545 cm^−1^. Increased absorbance at these wavelengths is due to the accumulation of C=O stretching, C–N stretching, and N–H bending, which together suggest an overall increase in tissue protein^47^ (Figure 1A). Similarly, there were overall increases in tissue glycogen levels shown by the increase in infrared absorbance in the peak mean centered at 1154 cm^−1^ and 1034 cm^−1^, which correlate with the COH deformation of glycogen and the CO–O–C asymmetric stretch of glycogen and nucleic acids, respectively^48^ (Figure 1A). Conversely, overall lipid signatures were reduced following cold acclimation (Table 3). These reductions were evident in the asymmetric and symmetric stretch of the CH_2_ and CH_3_ groups of lipids, at peaks centered at 2953 cm^−1^, 2874 cm^−1^, 2926 cm,^−1^ and 2855 cm^−1^, the ester C=O stretch of lipid, mean centered at 1742 cm^−1^, the CH_2_ bending of amino acid side chains of peptides and proteins, mean centered at 1451 cm^−1^, and stretching of amino acid residues at 1387 cm^−1^ ^49^ (Figure 1A). Additionally, there was a reduction in peaks at 1397 cm^−1^, 1240 cm,^−1^ and 1080 cm^−1^ suggesting reductions in the COO^−^ stretching of fatty acids and amino acids, and the asymmetric and symmetric stretching modes of nucleic acid. Principal component analysis (PCA) showed separation in the data by PC 1 and some separation along PC 2, explaining 72.1% and 15% of the variation, respectively (Figure 1B). PC loading plots revealed that the predominant spectral profiles separated by PC1 are those associated with higher levels of protein and glycogen and lower levels of lipid and phosphates (Figure 1C). The predominant spectral profiles in PC2 suggest that there were increases in amide A and glycogen and lactate absorption following cold acclimation (Figure 1C).

**TABLE 2:** The differences in infrared absorption between acclimation groups across key spectral bands.

Peak position (cm^−1^)	Assignment	Cold‐acclimated intensity	Control intensity	Difference
3069	Amide B	0.0192	0.0171	0.0021
2953	*ν*_as_CH_3_ of lipids, triglycerides, fatty acids, proteins	0.0358	0.0382	−0.0024
2924	*ν*_as_CH_2_ of lipids, triglycerides, proteins	0.0408	0.0455	−0.0047
2872	*ν*_s_CH_3_ of proteins, lipids, triglycerides	0.0231	0.025	−0.0019
2855	*ν*_s_CH_2_ of lipids, triglycerides, proteins	0.0205	0.023	−0.0025
1736	νC=O of triglycerides, cholesterol esters, phospholipids	0.0094	0.0127	−0.0033
1655	Amide I	0.113	0.101	0.012
1545	Amide II	0.0692	0.065	0.0042
1451	*δ*(CH_2_, CH_3_) of proteins, lipids	0.0286	0.0323	−0.0037
1387	*ν*_s_(COO^−^) free amino acids, fatty acids	0.0266	0.0293	−0.0027
1312	Amide III, erythrocytes	0.0172	0.019	−0.0018
1240	*ν*_as_ of nucleic acids, phospholipids, phosphorylated proteins, amide III	0.024	0.0276	−0.0036
1152	*ν*_as_(CO‐O‐C) of glycogen	0.0125	0.0106	0.0019
1086	*ν*_s_ of nucleic acids, phospholipids, phosphorylated proteins *ν*(CO) oligosaccharides, glycolipids	0.0305	0.0345	−0.004
1034	COH deformation glycogen	0.0237	0.02	−0.0037

*Note*: N.B. Difference = cold‐acclimated – control. Intensity = infrared absorption. Differences determined by PCA  (Figure 1).

**TABLE 3:** Differences in the infrared absorption profile of lipid with thermal acclimation.

Peaks	Equation	Cold‐acclimated ratio	Control ratio
Unsaturated lipids	*ν*(=CH)/*ν*_as_(CH_2_) + *ν*_s_(CH_2_)	9.277	8.72
Branched lipids	*ν*_as_(CH_3_)/*ν*_as_(CH_2_) + *ν*_s_(CH_2_)	0.259	0.118
Disorder of lipid acyl chains	*ν*_as_(CH_2_)/*ν*_s_(CH_2_)	7.818	7.612
Lipid to protein	*ν*(=CH)/amide I + amide II	0.322	0.433

The amide bands are composed of many narrower vibrational bands superimposed to make a broad band. Therefore, following analysis of mean spectra, we transformed the data into the second derivative to deconvolute these broad bands and identify specific differences in proteins. In cold‐acclimated animals, the second derivative of spectra showed an overall increase in protein with specific increases in two regions of the amide A band, 3302 cm^−1^ and 3270 cm^−1^, and in the amide B peak at 3067 cm^−1^ (Figure 1D). There were also changes in the spectra in 3 positions of the amide I band, 1692 cm^−1^, 1657 cm^−1^, and 1645 cm^−1^, and at two positions within the amide II band, 1547 cm^−1^ and 1514 cm^−1^ (Figure 1D). Interestingly, this deconvolution also revealed an increase in the band mean centered at 1338 cm^−1^, which is indicative of collagen amino acid side chain vibrations. The deconvoluted spectra also revealed increased glycogen and lactate levels, at 1154 cm^−1^ and 1029 cm^−1^, indicating an increase in glycolysis. In addition, there was a change in the asymmetric and symmetric stretching modes of phosphodiesterase of nucleic acid, at 1080 cm^−1^ (Figure 1D). Finally, the second derivative spectra suggested a decrease in overall tissue lipid following cold acclimation at peaks correlating to the asymmetric stretch of CH_2_, CH_3_, and the ester C=O stretch of lipids, at 2920 cm^−1^, 2852 cm^−1^, and 1742 cm^−1^. A decrease in the asymmetric stretch of PO_2_^−^, at 1235 cm^−1^, was also revealed (Figure 1D). Together, these results suggest a number of key differences in the tissue biochemistry of the turtle ventricle following thermal acclimation, particularly in areas of the spectrum correlating to signatures of proteins, lipids, and glycogen, and those associated with collagen.

### 3.2 Thermal acclimation regulates micromechanical tissue stiffness

**FIGURE 2:**
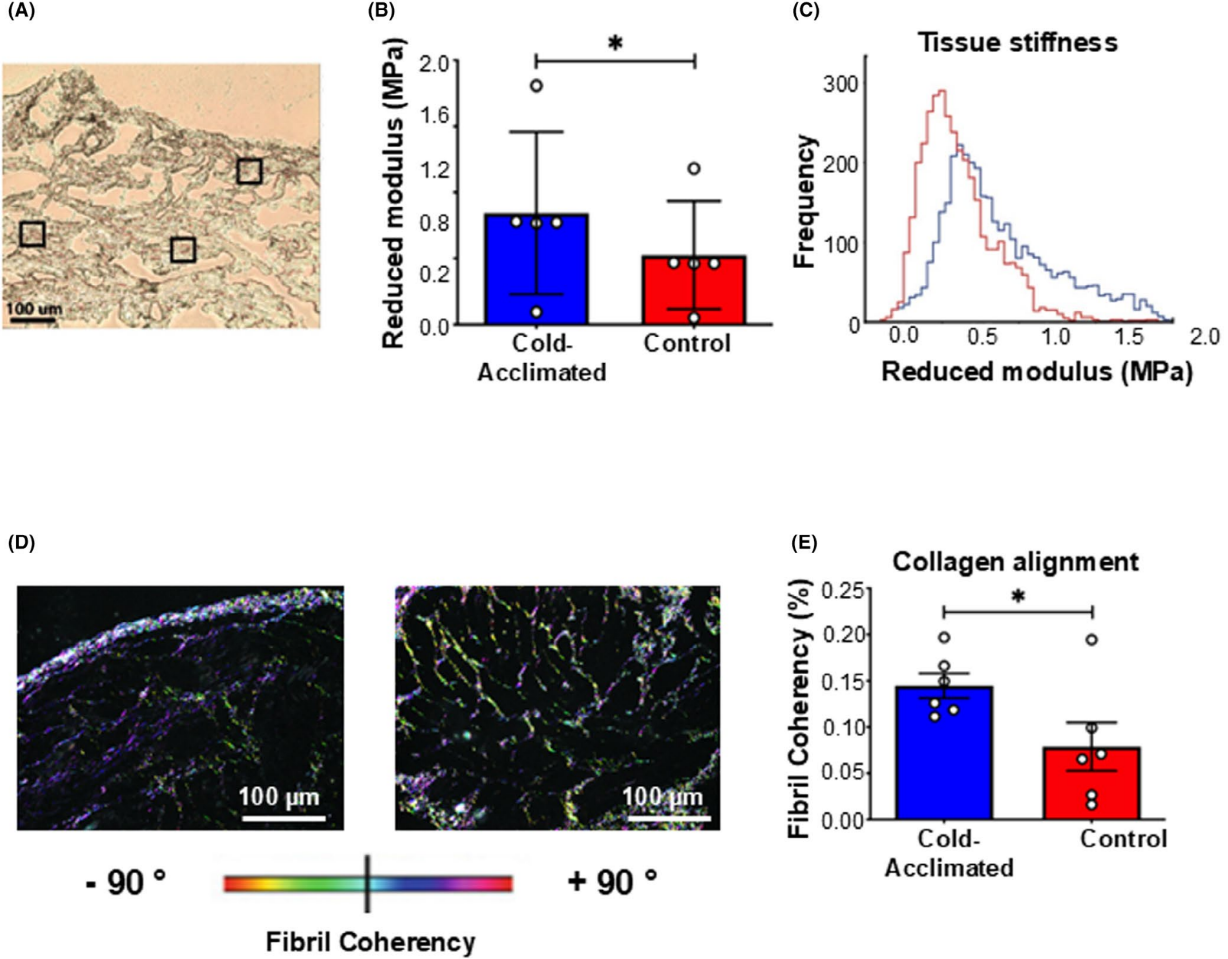
Micromechanics of ventricular tissue. (A) A brightfield image of a representative ventricular cryosection. The 3 square boxes show the 50 × 50 μm areas used for AFM nanoindentation. (B) The reduced modulus (M_r_). Values are presented as mean ± interquartile range and range. Individual dots are individual turtles. Significant differences in mean E_r_ between groups was assessed by GLM and indicated by * (*p* < 0.005) (C) Mean Er frequency curves calculated from 400 modulus measurements for each of three regions for *n* = 5 specimens in both cold‐acclimated (5°C; blue) and control (25°C; red) ventricular tissue (greater or less than 2 SD from the mean were discarded). (D) Representative polarized light micrographs for cold‐acclimated and control ventricular sections, stained with picro‐sirus red and assessed using OrientationJ. Fiber orientation is shown by color, with fibers of the same color showing coherency. (E) Quantification of coherency of organized fibrillar collagen for cold‐acclimated and control ventricular sections (*n* = 5). Values are presented as mean ± interquartile range and range. Individual dots are individual turtles. Significant differences in collagen coherency between groups were assessed by GLM and indicated by * (*p* < 0.05).

Following the FTIR results, which suggested a temperature‐dependent effect on tissue biochemistry, we next sought to determine the functional effect of temperature‐dependent cardiac remodeling. To assess the functional differences in the micromechanical stiffness of ventricular tissue sections, we used localized AFM nano‐indentation. Three 50 × 50 μm areas were tested on each tissue cryosection, shown in the representative control section, imaged under bright‐field light (Figure 2A). Nano‐indentation showed that the micromechanical stiffness of cold‐acclimated ventricular tissue was greater than that of control tissue (*p* < 0.001; Figure 2B). Mean reduced modulus (M_r_), and hence localized tissue stiffness, was correlated with temperature, giving higher and, therefore, stiffer values in the cold‐acclimated tissue compared with the control tissue. Although there was homogeneity in M_r_ between sample areas across the tissue as a whole, the reduced modulus frequency distribution within each sample area (Figure 2C) showed a shift in the peak of the frequency curve to the right (from ~0.4 MPa in control to ~0.6 MPa) following cold acclimation and a difference in the gradient at which the frequency curve tails off (i.e., an increased number of curves between 1.0–1.5 MPa). These results indicate that increased ventricular stiffness following temperature acclimation is not due to uniform structural and/or compositional remodeling of the tissue, but rather a tissue‐wide, but heterogeneous and localized, micro‐scale stiffening.

### 3.3 Thermal acclimation regulates fibrillar collagen content and coherency

Our FTIR results suggest changes in protein content of the tissue, and one specific protein that was shown to increase was collagen. This finding supports our previous work, where we showed that cold temperature acclimation increases collagen deposition in the turtle ventricle.^9^ However, the organization of collagen fibers is also important in determining overall tissue stiffness.^38^ We explored whether collagen alignment in the turtle ventricle was influenced by thermal acclimation by quantitatively measuring collagen coherency. Cold‐acclimated and control ventricular cryosections were stained with picro‐sirius red and imaged under polarized light before assessment using the OrientationJ plugin on ImageJ (Figure 2D,E). Quantitative analysis of fibrillar collagen coherency in turtle ventricle showed a 1.8‐fold increase in coherency of fibers following cold acclimation compared with controls (*p* < 0.05; Figure 2E).

**FIGURE 3:**
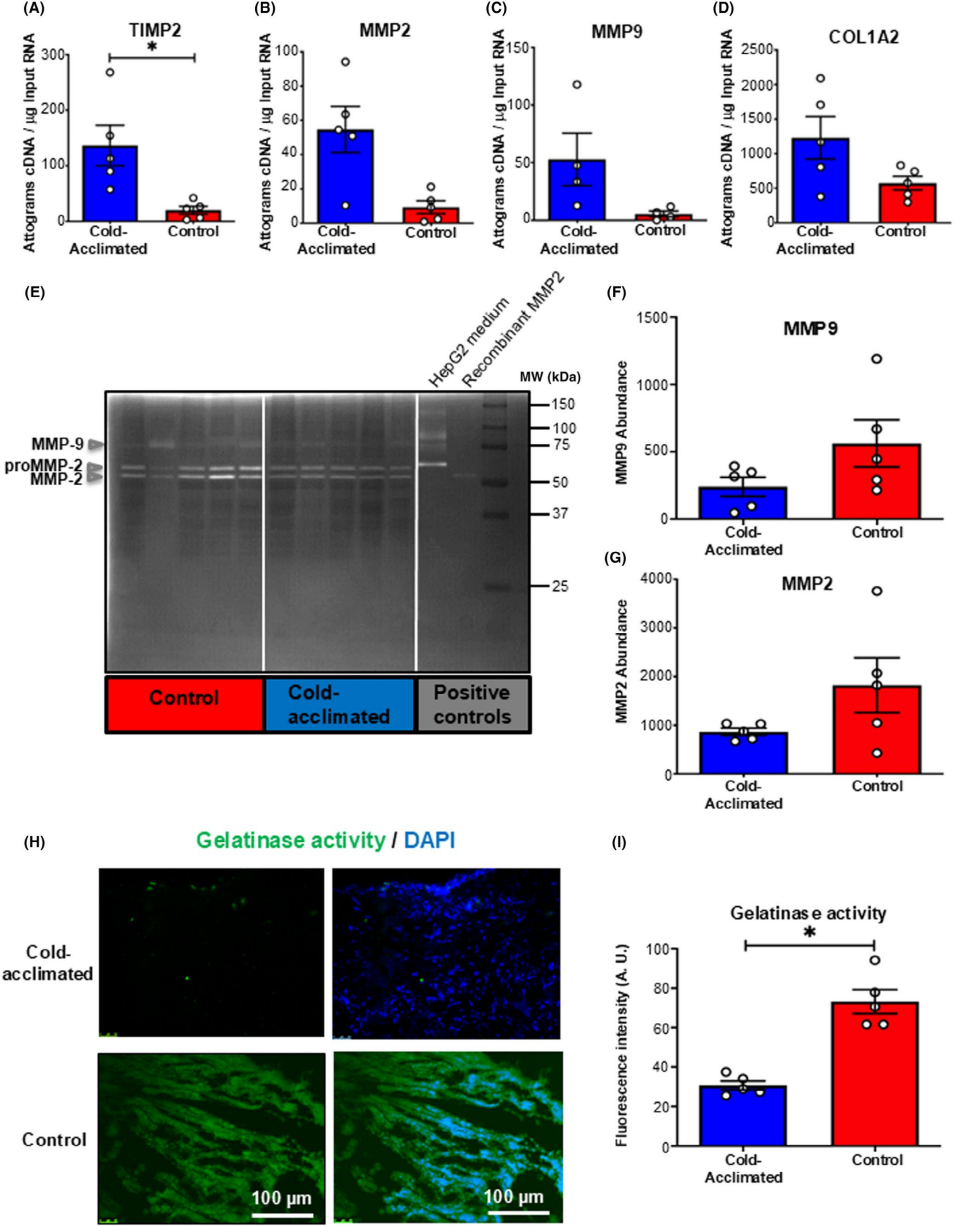
Regulation of ventricular connective tissue. (A–D) Quantitative real‐time PCR analysis of mRNA expression of (A) tissue inhibitor of matrix metalloproteinases (TIMP2), (B) collagen degrading matrix metalloproteinase‐2 (MMP2), (C) matrix metalloproteinase‐9 (MMP9), and (D) collagen I gene (COL1α2), for cold‐acclimated (5°C; striped) and control (25°C; filled) freshwater turtles (*n* = 5). Values are presented as mean ± *SE*. Individual dots are individual turtles. Significant differences in mRNA expression were assessed by GLM and indicated by * (*p* < 0.05). (E–G) Characterization of specific matrix metalloproteinase (MMP) activity in the ventricular myocardium by gelatin SDS‐PAGE zymography. (E) Coomassie R250 stained zymogram, indicating the relative molecular weights (MW, kDa) and abundances of gelatinases in ventricle extracts from cold‐acclimated (blue) and control (red) freshwater turtles (*n* = 5). The positions of MMP9, proMMP‐2 and MMP2 are indicated by arrows on the left‐hand side of the gel. (F) Abundance of MMP9 and (G) abundance of MMP2 from ventricle extracts of cold‐acclimated and control freshwater turtle (*n* = 5). Values are presented as mean ± *SE*. Individual dots are individual turtles. No significant differences in MMP abundance were found when assessed by GLM. (H, I) Endogenous matrix metalloproteinase (MMP) activity. (H) Representative fluorescent micrographs for cold‐acclimated and control ventricle imaged with a green filter to show gelatinase activity. The same sections were imaged in with a blue filter and the image imposed to show DAPI fluorescence for cold‐acclimated and control turtle tissue. (I) Semi‐quantitative analysis of endogenous MMP activity by in situ gelatinase zymography of ventricular sections for cold‐acclimated and control freshwater turtles (*n* = 5). Values are presented as mean ± interquartile range and range. Individual dots are individual turtles. Significant differences in collagen coherency were assessed by GLM and indicated by * (*p* < 0.05).

We next sought to determine a mechanism for this temperature‐dependent collagen regulation. Matrix metalloproteinases (MMPs) regulate collagen degradation in the ECM.^50^ We used RT‐qPCR to assess whether there was a temperature‐dependent change in gene expression of collagen synthesis and degradation‐regulating proteins (Figure 3A–D). Temperature acclimation did not significantly alter mRNA expression of the collagen I gene (COL1α2), MMP2, or MMP9 despite a trend for increased expression in the cold compared to control (Figure 3B–D). However, MMP activity is more complex than transcription alone. One mechanism for regulating MMP activity at the tissue level is via tissue inhibitors of metalloproteinases (TIMPs)^50^ and we found a 6.9‐fold increase in the mRNA expression of TIMP2 following cold acclimation compared to controls (*p* < 0.05; Figure 3A).

We next wanted to assess whether TIMP2 could be regulating MMP protein levels and activity, thereby driving the temperature‐induced changes in collagen deposition. To assess whether temperature acclimation altered MMP protein levels, we used gelatin SDS‐PAGE zymography. MMPs are initially synthesized as inactive pro‐forms, and then the pro‐domain must be removed to activate the enzyme.^51^ Zymographs showed the presence of MMPs with molecular weights matching those of human‐activated MMP‐9 and human proMMP‐2, and human‐activated MMP‐2 in the turtle ventricle (Figure 3E–G). We did not find any significant differences in the abundance of proMMP‐2, MMP2, or MMP9, nor in the ratio of proMMP‐2 to MMP2, following cold acclimation compared to controls; however, there was a trend towards decreased abundance of MMP2 and MMP9 following cold acclimation (Figure 3E–G). Although this suggests MMP *levels* are unchanged, TIMP2 may be affecting MMP *activity*. Therefore, we tested the endogenous gelatinase activity of MMPs by in situ zymography. Cold‐acclimated and control turtle ventricle cryosections were coated with DQ gelatin, and localized activity of MMPs is revealed by the corresponding green fluorescent signal (Figure 3A,B). Semi‐quantitative analysis of fluorescent micrographs showed a 1.7‐fold lower gelatinase activity of MMPs following cold acclimation compared to controls (*p* < 0.005; Figure 3C). Together, these results suggest that the increased collagen content in cold‐acclimated turtle ventricle is due, at least in part, to an increase in the expression of TIMP2, which subsequently suppresses MMP activity.

### 3.4 Effects of cold acclimation on metabolic substrate utilization

**FIGURE 4:**
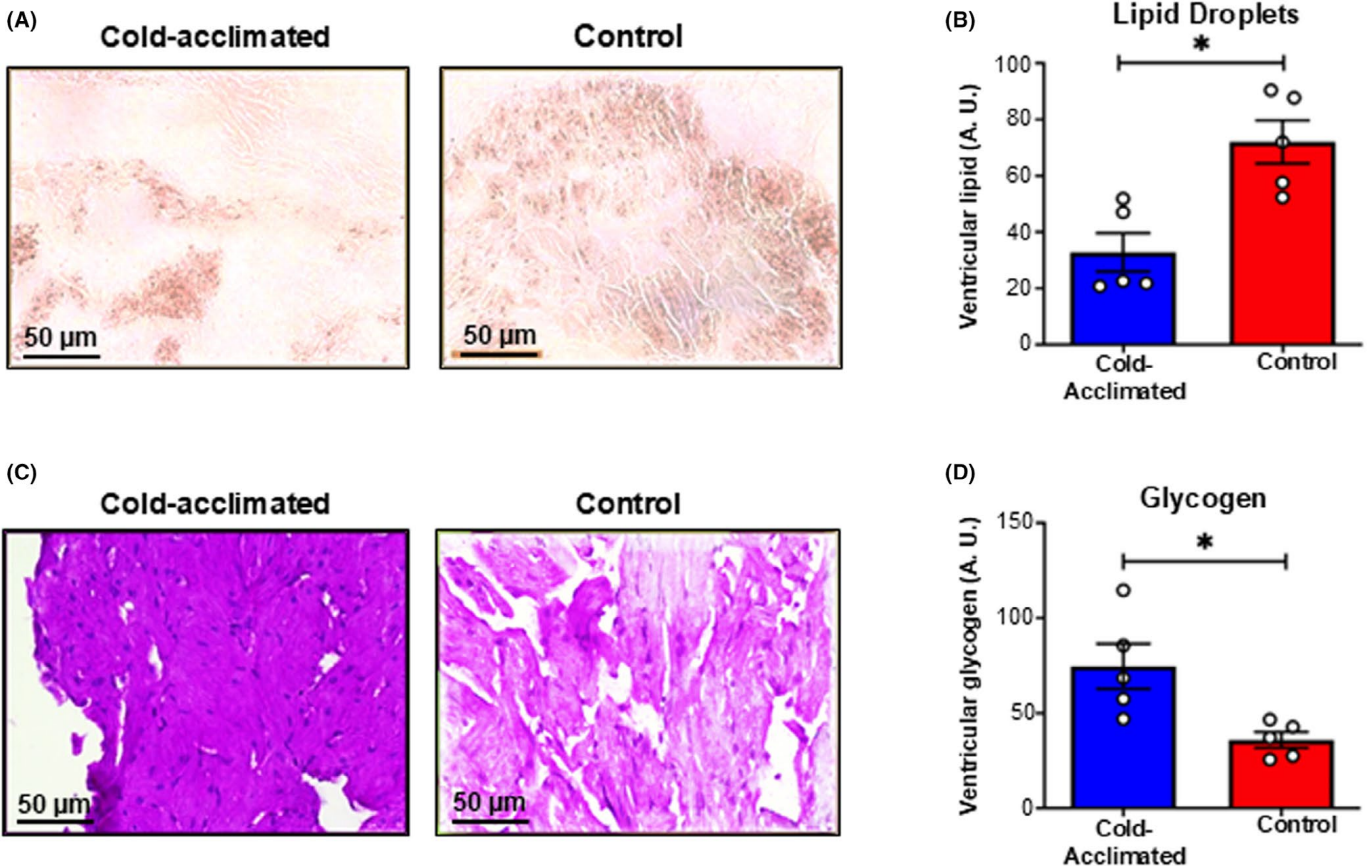
Remodeling of metabolic substrates with cold acclimation. Histological staining for lipid and glycogen in the turtle ventricle. A representative micrograph of (A) cold‐acclimated (5°C) and control (25°C) turtle ventricular cryosections stained with oil red‐O, which stains lipid droplets red. (B) Semi‐quantitative analysis of oil red‐O staining for cold‐acclimated (blue) and control (red) turtle ventricle cryosections (*n* = 5 for each group). (C) A representative micrograph of cold‐acclimated and control turtle formalin‐fixed ventricular sections stained with periodic acid Schiff (PAS), which stains glycogen purple. (D) Semi‐quantitative analysis of PAS staining for cold‐acclimated and control turtle ventricle cryosections (*n* = 5 for each group). Values are presented as mean ± *SE*. Individual dots are individual turtles. Significant differences between acclimation groups were determined by GLM and indicated by *.

Our FTIR results indicated differences in lipid and glycogen profiles following thermal acclimation. We next substantiated this finding using a histology and biochemical approach. The lipid content of the turtle ventricle was assessed using oil red‐O stain, which stains lipid droplets in red (Figure 4A). We found a 2.2‐fold decrease in oil red‐O stain following cold acclimation (Figure 4A,B). To assess glycogen content, we used PAS stain, which selectively stains glycogen in purple (Figure 4C). We found cold acclimation increased purple stain in the tissue by 2.9‐fold compared with controls (Figure 4C,D). Finally, we also assessed the glycogen content of the ventricle using a biochemical assay. Here we saw no difference in glycogen content between control and cold‐acclimated tissue (control = 0.325 ± 0.027; cold acclimated = 0.351 ± 0.017 μmol glycosyl unit/mg protein, *n* = 5, not shown).

## 4 DISCUSSION

Dynamic fibrotic cardiac remodeling is critical for freshwater turtles to survive prolonged cold winters characterized by reduced activity and hypometabolism. Hearts from turtles living in warm conditions do not show the same fibrotic morphology.^9^ This observation has received interest from comparative physiologists and cardiac physiologists as it presents a potential model for reversible remodeling with applications to biomedicine.^14^, ^21^, ^22^, ^52^, ^53^ Here, we provide a comprehensive assessment of the structural remodeling response in *T. scripta* hearts to look for parallels with human pathological fibrosis. We also show metabolic substrate alteration suggesting an adaptive response in fuel deposition facilitating the cold‐induced switch in cardiac energetics. We conclude that extracellular matrix remodeling and alteration in MMPs and their inhibitors are important for stabilizing cardiac structure during remodeling. Changes in metabolism also accompany the structural remodeling response in humans (dysregulation) and turtles (suppression). Thus, cold‐induced fibrosis in turtles can contribute to our understanding of how ECM remodeling protects the heart without long‐term detriment. This knowledge might inform strategies to mitigate or reverse pathological fibrosis in humans.

### 4.1 Regulation of myocardial collagen with thermal acclimation

FTIR analysis showed an increase in overall protein content of the ventricle and a decrease in the lipid‐to‐protein ratio following cold acclimation (Table 3). Following data deconvolution, by converting to the second derivative, it was possible to determine peaks that suggest different types of secondary conformations. We saw changes in bands associated with anti‐parallel turns (1692 cm^−1^), α‐helices (1657 cm^−1^) and β‐sheet conformations (1645 cm^−1^).^54^, ^55^ Collagen is a complex fibrillar molecule, and therefore, there are numerous spectral bands that have been assigned to collagen, including 1206 cm^−1^, 1238 cm^−1^, and 1280 cm^−1^.^56^, ^57^ As the turtle myocardium has relatively low levels of collagen under normal conditions, and due to artifacts of converting the spectra to the second derivative, it was difficult to fully resolve these spectral bands, but we did see a clear change at 1202 cm^−1^. Cheheltani et al.^57^ showed that collagen can be resolved well by the peak at 1338 cm^−1^, which arises due to specific collagen amino acid side chains, and we also saw an increase in this band following cold acclimation. This agrees with our previous work showing an increase in ventricular collagen content following cold acclimation.^9^

As fibrillar collagen is responsible for stiffness in the myocardium,^58^ tight and precise regulation is critical for correct cardiac function.^50^, ^51^ Thus, the regulation of deposition and degradation of the ECM proteins must be altered by cold acclimation. MMPs are important regulators of tissue homeostasis involved in cellular signaling pathways and ECM degradation; therefore, their activity is closely regulated across multiple biological levels.^50^, ^51^, ^59^ We did not find any changes in the mRNA expression of the collagen I gene (COL1α2) or either of the MMP genes we tested. However, there was a trend towards increased expression of COL1α2, MMP2, and MMP9 with cold acclimation. This trend may be explained by the involvement of MMPs in degrading components of the ECM so that remodeling can occur.^60^, ^61^, ^62^ Despite no change in gene expression, we did see a decrease in the endogenous gelatinase activity of MMPs following cold acclimation, suggesting less degradation of collagen fibrils.^40^, ^63^ A change in MMP activity may be due to a decrease in mRNA translation or a decrease in post‐translational activation of MMPs in the tissue, i.e., a change in the ratio of proMMP to activated MMP. Indeed, our gelatin zymography result would support this, showing a trend towards higher levels of pro‐MMP2, MMP2, and MMP9 in control tissue.

We report a significant difference in the expression of TIMP2 mRNA, which was increased following cold acclimation, suggesting up‐regulation of this gene. TIMP2 inhibits the collagen‐degrading activity of MMPs which is in agreement with the reduced MMP activity we observed in our in situ zymography results, leading to an increase in tissue collagen content.^51^ Interestingly, as changes in ambient temperature occur seasonally, it is likely that this phenotype is plastic and switches as temperatures rise during the spring and summer months (see^64^). Therefore, regulation of tissue ECM proteins may be crucial to understanding cardiac remodeling in freshwater turtles.

### 4.2 Increases in the micromechanical stiffness of ventricular tissue

We have previously shown that chronic cold causes fibrosis of the turtle ventricle.^9^ Collagen is an important mediator of tissue tensile strength and stiffness and is arranged into networks that support cardiomyocytes^65^ and impact cardiac micromechanical properties.^65^ Here, we use AFM nano‐indentation to reveal an increase in micromechanical stiffness across the whole of the ventricular tissue with chronic cold. Particularly interesting were the changes in the accumulative frequency curve of reduced modulus (M_r_) which suggest an increase in the heterogeneity of the tissue following cold acclimation. This, in turn, suggests stiffness is modulated by the density of stiff fibers in the tissue. It is important to note that this increased heterogeneity was observed within each sample area, rather than between sample areas, suggesting that this change was homogeneous across the tissue section rather than a differential remodeling of stiffness in discrete layers of cardiac tissue. The nano‐scale resolution of AFM means the contribution of smaller perimysial collagen to overall tissue stiffness is also measured.^66^ We found a high frequency of force curves with a M_r_ between 1.0 and 1.5 MPa, which is consistent with M_r_ of tissue with high collagen fiber content indented at a lower loading rate.^38^, ^67^, ^68^ Therefore, it appears that the stiffness of the turtle ventricular myocardium following thermal acclimation is modulated, at least in part, by the relative content of fibrillar collagen.

We also assessed the coherency in alignment of collagen fibers as recent studies suggest that organization of collagen fibers can affect overall tissue stiffness.^38^ We used coherency of collagen fibrils to assess collagen alignment, with coherency specifically assessing the percentage of collagen fibrils at the same angle.^37^ These changes in fibrillar collagen alignment, along with changes in fibrillar collagen content,^9^ can be used to explain increases in tissue stiffness with prolonged cold.^38^ Future work could investigate the impact of the thermal environment on collagen cross‐linking as it is also thought to impact matrix stiffness.

### 4.3 Changes in metabolic substrate following cold acclimation

The heart requires a continuous and sufficient supply of ATP, which under normal conditions is predominantly achieved by mitochondrial FAO.^69^ Although a shift in cardiac energetics towards glycolysis is a hallmark of pathology in the human heart,^69^, ^70^, ^71^ turtles are known to use this to their advantage during winter metabolic suppression.^14^, ^20^, ^21^, ^72^ Following cold acclimation, we saw an overall decrease in the spectral profile of triglycerides and cholesterol esters. The CH stretching region of the spectrum (from ~2700 to 3100 cm^−1^) can be used to estimate overall lipid content^73^ and we show absorption in this region was decreased following cold acclimation. This is a complex region of the spectrum created by a large number of nearly identical vibrations, superimposed to form a broad band. Within the broad band, there are two regions characteristic of saturated long chain hydrocarbons, showing the symmetric and asymmetric stretching modes of CH_2_ (~2926 cm^−1^ and 2855 cm^−1^).^73^, ^74^ There is another pair of distinct regions which show the symmetric and asymmetric stretching modes of CH_3_ (2953 cm^−1^ and 2874 cm^−1^).^73^ The ratio between the asymmetric stretch of CH_3_ and the asymmetric stretch of CH_2_ can be used to determine the degree of lipid saturation in the tissue, with a higher absorption of CH_2_ indicating a higher number of C=C double bonds.^54^, ^73^ Following cold acclimation, the ratio of lipid unsaturation was increased (Table 3). From this region, it is also possible to determine the ratio of methyl to methylene groups and, therefore, the degree of branching of lipids.^54^, ^73^ Our data suggest a higher tissue content of branched rather than long chain fatty acids following cold acclimation (Table 3).^54^ Lastly, it was possible to assess the state of biological membranes by the ratio of asymmetric to symmetric CH_2_ stretch.^54^ Following cold acclimation, we found that the disorder of lipid acyl chains also increased (Table 3). We also saw a decrease in the C=O stretching of cholesterol esters and triglycerides (~1740 cm^−1^). Due to the difference in shape of the shoulder, it is likely that the difference was due to a reduction in tissue cholesterol esters rather than a decrease in triglycerides as the carbonyl stretching for triglycerides is more typically ~1745 cm^−1^, at which point the spectra are closer.^54^ However, this band is susceptible to rapid oxidation once cryosections have thawed.^75^ To counteract this, we ensured that samples were imaged quickly after removal from the freezer and within a timeframe that oxidation is minimal.^75^ Although we cannot rule out the possibility that oxidation may have affected our data in this wavenumber region, the reduction in ventricular lipid storage was supported by histological assessment of lipid content by oil red‐O staining.

The mean spectral profiles correlating to glycogen show an increased infrared absorption at 1152 cm^−1^ and 1034 cm^−1^, which are indicative of glycogen, glycolipids, and glycoproteins.^54^, ^76^, ^77^, ^78^ In addition, following the conversion of the mean spectra to the second derivative, the deconvolution of the spectra allowed resolution of peaks at 1080 cm^−1^ and 1029 cm^−1^. These peaks also showed differences with thermal acclimation and are indicative of glycolysis, suggesting differences in glycogen and lactate (a metabolic by‐product of anaerobic glycolysis) use.^78^ Histological PAS staining agreed with the FTIR result, showing increased levels of glycogen following cold acclimation (Figure 4C,D). However, tissue glycogen content was unchanged when assessed via biochemical assay. We are not able to explain why the changes detected with the FTIR and histological approaches were not substantiated by the biochemical assay. An increase in ventricular glycogen with cold acclimation has been observed previously in *T. Scripta* using biochemical assays.^79^ Similar reductions in FAO and increases in glycogen have been observed in cold‐acclimated turtle hearts assessed via metabolomics,^72^ with the authors supporting the idea that FAO provides a more energy‐dense fuel source compared to glycogen in the warm, which is not required when they are inactive in the cold. Indeed, metabolic shifts are a common response in seasonal ectothermic species.^80^, ^81^

### 4.4 Changes in phosphate macromolecules following thermal acclimation

Following cold acclimation, we saw decreases in the infrared absorption of bands at ~1086 cm^−1^ and 1240 cm^−1^, which are where the main symmetric and asymmetric phosphate group vibrations appear.^82^ These bands contain the vibrations of phosphate molecules in DNA, RNA, phospholipids, and phosphorylated proteins,^78^, ^83^, ^84^, ^85^, ^86^, ^87^ which makes it difficult to determine the contribution of each molecule. It is also possible that if ATP stores are depleted due to higher myocardial ATP demand than supply during glycolysis, the reduced ATP molecules would influence this spectral region as was found using ^31^P‐NMR spectroscopy.^88^

## 5 CONCLUSIONS

In this study, we have probed multiple aspects of the adaptive remodeling response of the turtle ventricle to prolonged cold temperature. We show increased connective tissue and changes in collagen regulatory proteins that contribute to the increased tissue stiffness. We reveal an overall decrease in ventricular lipids and phosphates following cold acclimation and an increase in glycogen and products of glycolysis, such as lactate. Together, our results suggest that prolonged exposure to cold primes the ventricle for a metabolic shift away from FAO and toward glycolysis. It is likely that the metabolic shift from FAO to glycolysis is an early cardio‐protective event readying the heart for the subsequent onset of winter hypoxia under ice‐covered ponds where reduced oxygen demand is required for survival. This metabolic shift has correlates in the metabolic dysregulation that is known to contribute to cardiac fibrosis in human cardiac pathologies.^24^ As seasonal remodeling in turtle hearts is a well‐known phenomenon,^89^, ^90^, ^91^ we hypothesize that the mechanical and energetic cold remodeling of the turtle heart reverts back to reduced stiffness and increased reliance on FAO upon ambient warming in spring. To experimentally show reversibility, future studies could employ a non‐invasive technique like ^31^P‐NMR spectroscopy or MRI^92^ to assess thermal remodeling in vivo during chronic cooling and rewarming. Such studies are necessary to determine the utility of the turtle heart as a model for the reversibility of micromechanical and energetic remodeling associated with human cardiomyopathies.

## AUTHOR CONTRIBUTIONS

**Adam N. Keen:** Formal analysis; investigation; visualization; Writing – original draft. **James C. McConnell:** Formal analysis; investigation. **John J. Mackrill:** Investigation; formal analysis; methodology. **John Marrin:** Investigation; formal analysis. **Alex J. Holsgrove:** Investigation; formal analysis. **Janna Crossley:** Investigation; formal analysis. **Alex Henderson:** Methodology; supervision. **Gina L. J. Galli:** Methodology; supervision. **Dane A. Crossley II:** Project administration; resources; supervision. **Michael J. Sherratt:** Conceptualization; methodology; project administration; supervision. **Peter Gardner:** Conceptualization; funding acquisition; methodology; project administration; supervision. **Holly A. Shiels:** Conceptualization; funding acquisition; methodology; project administration; supervision; writing – review and editing.

## CONFLICT OF INTEREST STATEMENT

The authors declare that they have no known competing financial interests or personal relationships that could have appeared to influence the work reported in this paper.

## Supporting information


Data S1.

## Data Availability

The data that support the findings of this study are available on request from the corresponding author. The data are not publicly available due to privacy or ethical restrictions.
