# Supplementary material for: Cold‐induced fibrosis and metabolic remodeling in the turtle (Trachemys scripta) ventricle
Source: Acta Physiol (Oxf). 2025 Mar 15;241(4):e70026. doi: 10.1111/apha.70026 (PMC11909586; doi:10.1111/apha.70026)
Supplement: Supplementary file 1 — Data S1. [file APHA-241-e70026-s001.pptx]

## Slide 1
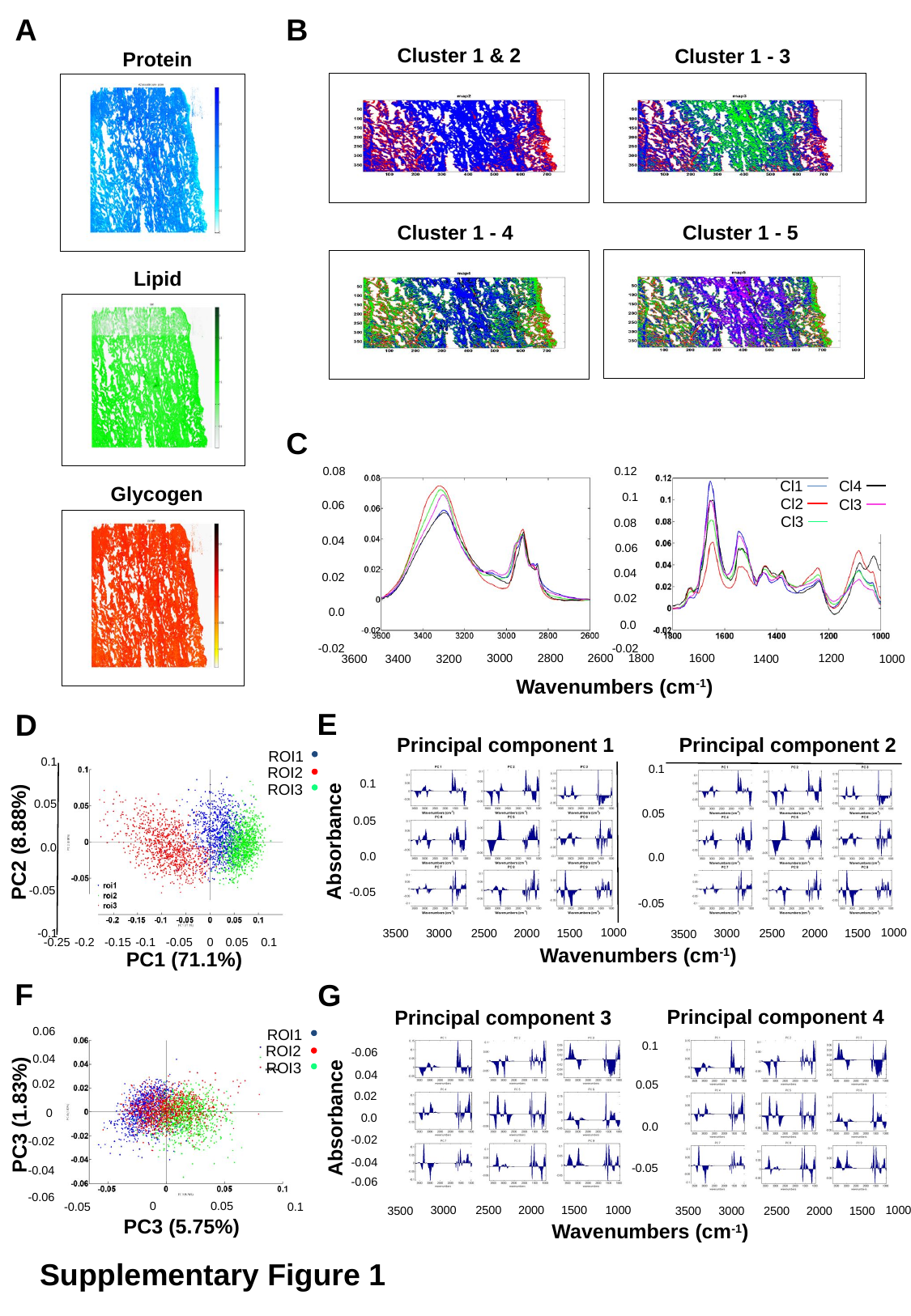

A
B
Cluster 1 & 2
Cluster 1 - 3
Protein
Cluster 1 - 4
Cluster 1 - 5
Lipid
C
0.08
0.12
Cl4
Cl1
Glycogen
0.1
Cl2
Cl3
0.06
Cl3
0.08
0.04
0.06
0.04
0.02
0.02
0.0
0.0
-0.02
-0.02
3000
2800
2600
1600
1200
1800
3600
3400
3200
1400
1000
Wavenumbers (cm-1)
E
D
Principal component 2
Principal component 1
ROI1
0.1
0.1
ROI2
0.1
ROI3
0.05
0.05
0.05
Absorbance
PC2 (8.88%)
0.0
0.0
0.0
-0.05
-0.05
-0.05
1000
1000
3000
2500
2000
1500
3500
3000
2500
2000
1500
-0.1
3500
-0.25
-0.2
-0.15
-0.1
-0.05
0
0.05
0.1
Wavenumbers (cm-1)
PC1 (71.1%)
F
G
Principal component 4
Principal component 3
0.06
ROI1
0.1
ROI2
-0.06
0.04
ROI3
0.04
0.02
0.05
0.02
PC3 (1.83%)
Absorbance
0
0.0
0.0
-0.02
-0.02
-0.04
-0.05
-0.04
-0.06
-0.06
0
0.05
-0.05
0.1
1000
1000
3000
2500
2000
1500
3500
3000
2500
2000
1500
3500
PC3 (5.75%)
Wavenumbers (cm-1)
Supplementary Figure 1

## Slide 2
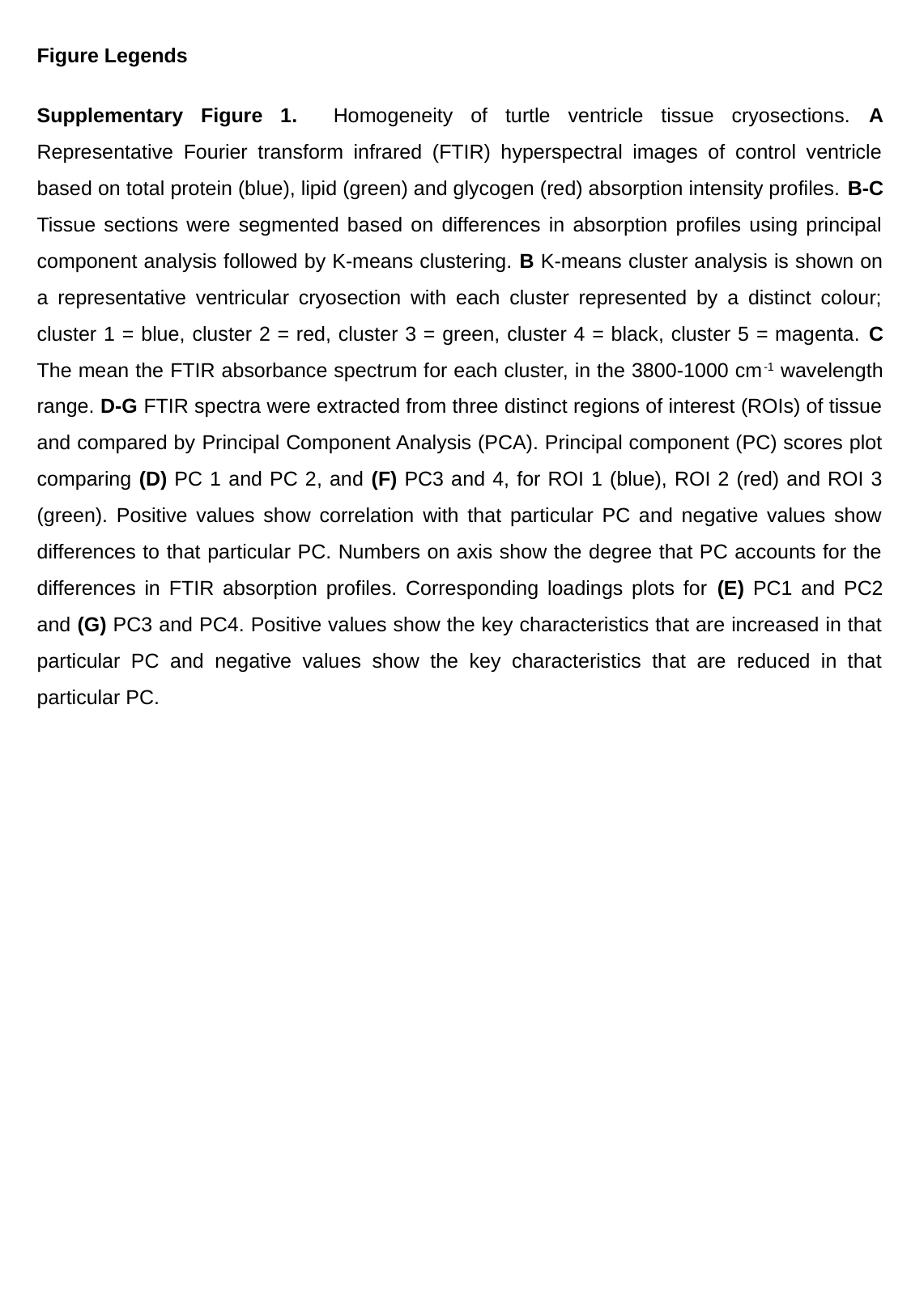

Figure Legends
Supplementary Figure 1. Homogeneity of turtle ventricle tissue cryosections. A Representative Fourier transform infrared (FTIR) hyperspectral images of control ventricle based on total protein (blue), lipid (green) and glycogen (red) absorption intensity profiles. B-C Tissue sections were segmented based on differences in absorption profiles using principal component analysis followed by K-means clustering. B K-means cluster analysis is shown on a representative ventricular cryosection with each cluster represented by a distinct colour; cluster 1 = blue, cluster 2 = red, cluster 3 = green, cluster 4 = black, cluster 5 = magenta. C The mean the FTIR absorbance spectrum for each cluster, in the 3800-1000 cm-1 wavelength range. D-G FTIR spectra were extracted from three distinct regions of interest (ROIs) of tissue and compared by Principal Component Analysis (PCA). Principal component (PC) scores plot comparing (D) PC 1 and PC 2, and (F) PC3 and 4, for ROI 1 (blue), ROI 2 (red) and ROI 3 (green). Positive values show correlation with that particular PC and negative values show differences to that particular PC. Numbers on axis show the degree that PC accounts for the differences in FTIR absorption profiles. Corresponding loadings plots for (E) PC1 and PC2 and (G) PC3 and PC4. Positive values show the key characteristics that are increased in that particular PC and negative values show the key characteristics that are reduced in that particular PC.
